# Corilagin Alleviates Nonalcoholic Fatty Liver Disease in High-Fat Diet-Induced C57BL/6 Mice by Ameliorating Oxidative Stress and Restoring Autophagic Flux

**DOI:** 10.3389/fphar.2019.01693

**Published:** 2020-02-04

**Authors:** Rong Zhang, Kexin Chu, Nengjiang Zhao, Jingjing Wu, Lina Ma, Chenfang Zhu, Xia Chen, Gang Wei, Mingjuan Liao

**Affiliations:** ^1^Department of Endocrinology and Metabolism, Shanghai General Hospital, Shanghai Jiao Tong University School of Medicine, Shanghai, China; ^2^Department of Radiation Oncology, Xiamen Cancer Hospital, The First Affiliated Hospital of Xiamen University, Xiamen, China; ^3^Department of Traditional Chinese Medicine Studio, The First Affiliated Hospital of Xiamen University, Xiamen, China; ^4^Department of Breast, Longhua Hospital, Shanghai University of Traditional Chinese Medicine, Shanghai, China; ^5^Department of General Surgery, The Ninth People’s Hospital, Medical School of Shanghai Jiaotong University, Shanghai, China; ^6^Department of Endocrinology and Metabolism, Shanghai Fourth People’s Hospital Affiliated to Tongji University School of Medicine, Shanghai, China; ^7^Shanghai Key Laboratory of Diabetes, Shanghai Institute for Diabetes, Shanghai Clinical Medical Centre of Diabetes, Shanghai Key Clinical Centre of Metabolic Diseases, Department of Endocrinology and Metabolism, Shanghai Jiao Tong University Affiliated Sixth People’s Hospital, Shanghai, China; ^8^Department of Traditional Chinese Medicine, The Ninth People’s Hospital, Medical School of Shanghai Jiaotong University, Shanghai, China

**Keywords:** Corilagin, nonalcoholic fatty liver disease, autophagy, oxidative stress, mitochondrial dysfunction

## Abstract

Corilagin (Cori) possesses multiple biological activities. To determine whether Cori can exert protective effects against nonalcoholic fatty liver disease (NAFLD) and its potential mechanisms. C57BL/6 mice were fed with high-fat diet (HFD) alone or in combination with Cori (20 mg/kg, i.p.) and AML12 cells were exposed to 200 μM PA/OA with or without Cori (10 μM or 20 μM). Phenotypes and key indicators relevant to NAFLD were examined both *in vivo* and *in vitro*. In this study, Cori significantly ameliorated hepatic steatosis, confirmed by improved serum lipid profiles, and hepatic TC, TG contents, and the gene expression related to lipid metabolism in livers of HFD mice. Moreover, Cori attenuated HFD-mediated autophagy (including mitophagy) blockage by restoring autophagic flux, evidenced by increased number of autophagic double vesicles containing mitochondria, elevated LC3II protein levels, decreased p62 protein levels, as well as enhanced colocalization of autophagy-related protein (LC3, Parkin) and mitochondria. In accordance with this, Cori also reduced the accumulation of ROS and MDA levels, and enhanced the activities of antioxidative enzymes including SOD, GSH-Px, and CAT. In addition, Cori treatment improved mitochondrial dysfunction, evidenced by increased mitochondrial membrane potential (ΔΨm). In parallel with this, Cori decreased mitochondrial DNA oxidative damage, while increased mitochondrial biogenesis related transcription factors expression, mitochondrial DNA content and oxygen consumption rate (OCR). In conclusion, these results demonstrate that Cori is a potential candidate for the treatment of NAFLD *via* diminishing oxidative stress, restoring autophagic flux, as well as improving mitochondrial functions.

## Introduction

Nonalcoholic fatty liver disease (NAFLD) has emerged as epidemic trend worldwide, which affects 6%–35% of adults globally (Sayiner et al., 2016; Bellentani, 2017; Cobbina and Akhlaghi, 2017). NAFLD is associated with energy metabolism dysfunction, which is mainly characterized by excess lipid deposition inside the liver parenchyma. NAFLD is the most common cause of chronic liver disease and can result in serious liver-related complications, thereby leading to an increase in overall mortality (Onnerhag et al., 2019). NAFLD encompasses a broad clinicopathological spectrum that ranges from simple steatosis to the more aggressive nonalcoholic steatohepatitis (NASH), with some people even ultimately progressing to fibrosis, cirrhosis, and liver cancer (Wree et al., 2013). Even though the simple steatosis is reversible and relatively benign, pharmacological therapy established for the treatment of NAFLD is far from meeting the clinical needs.

As an integral part of metabolic syndrome, NAFLD often occurs with other metabolic disorders (e.g., obesity and diabetes). The “two hits” hypothesis is now widely accepted, whereas, up to now, the pathogenesis of NAFLD has not yet been fully illuminated. The “first hit” is excessive lipid accumulation in the liver parenchyma cells caused by insulin resistance. The “second hit,” caused by the first hit, involves a cytotoxic reaction, such as oxidative stress and inflammatory responses (Day and James, 1998). Oxidative stress is a critical component of harmful cascades which is activated through the overproduction of ROS in the development of NAFLD (Oliveira et al., 2002; Lee et al., 2019). Mitochondria are the main site involved in fatty acid metabolism and reactive oxygen species (ROS) production (Scherz-Shouval and Elazar, 2011). During NAFLD, excessive lipid accumulation and elevated ROS levels may trigger oxidative stress in the hepatocytes, which may further disrupt mitochondrial architecture and subsequently impair mitochondrial function (Wang et al., 2015). In turn, defective mitochondrial activity leads to inadequate capacity of mitochondrial fatty acid oxidation and progressively accelerates ROS-mediated mitochondria dysfunction, thus contributing to the accumulation of lipid droplets in the liver and eventually causing hepatic steatosis (Nassir and Ibdah, 2014).

Autophagy is a self-degradative process, whereby intracytoplasmic components are delivered to lysosomes to be degraded for maintaining cellular homeostasis under physiological conditions, and can be activated as an adaptive catabolic process in response to metabolic stress (Mizushima and Levine, 2010; Czaja et al., 2013). Studies have established the essential roles of basal autophagy in the turnover of intracellular organelles, degradation of long-lived cytosolic proteins and damaged proteins (Czaja et al., 2013). Thereby, the contribution of induced autophagy is essential to cell survival by supplying amino acids, glucose and energy, which plays a key role in maintaining the cellular integrity during metabolic stress (Levine and Kroemer, 2008).

Accumulating data point out a pivotal role of ROS in the initiation of autophagy (Sinha et al., 2015). Despite this, overproduction of ROS continuously attacks mitochondria, which may cause mitochondrial fatigue and mitochondrial decompensation, leading to exacerbation of lipid overload and lipotoxicity, and eventually blocks the autophagic flux (Wang et al., 2015). Recently, it has been demonstrated that inhibition of autophagy resulted in significant accumulation of intracellular lipids, which was confirmed by the established hepatocyte-specific autophagy gene knockout mouse model (Singh et al., 2009). Correspondingly, there is a self-perpetuating vicious cycle between lipid deposition and autophagy suppression in the development and progression of NAFLD.

Natural products could provide new clues to pharmacological research and discovery of NAFLD. Previous studies have indicated that some Chinese herbal extracts (e.g., curcumin, resveratrol, and rutin) help to prevent the progression of NAFLD *in vivo* and *in vitro* (Shang et al., 2008; Yuan et al., 2017; Cicero et al., 2019). To date, potential mechanisms attributing to NAFLD are still unclear, thus developing new specific therapeutics for NAFLD are urgently needed.

**Figure 1:**
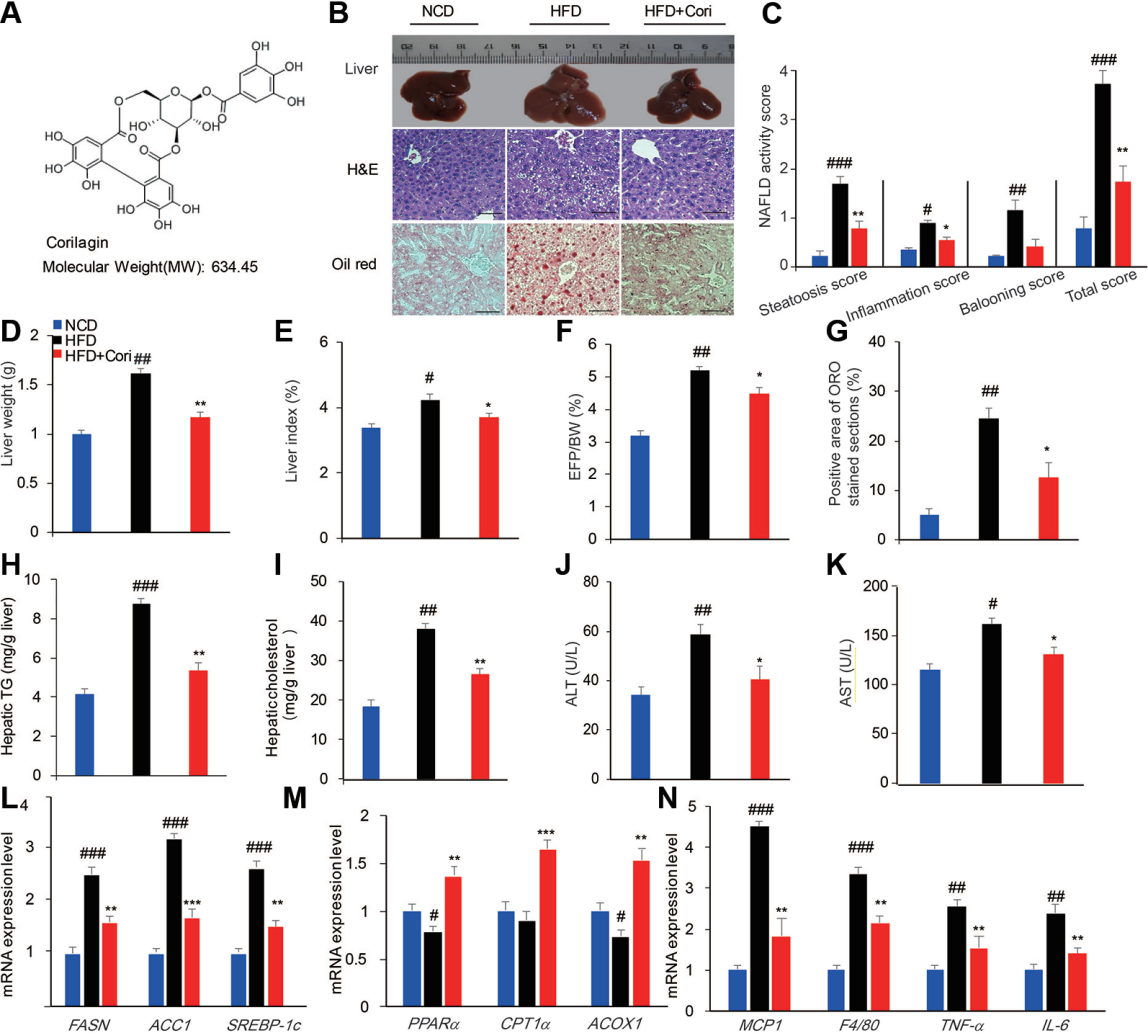
Cori ameliorates hepatic lipid accumulation in livers of high-fat diet (HFD)-induced C57BL6 mice. 6-week-old male C57BL/6 mice (n = 30) were fed with normal chow diet (normal chow diet [NCD] group, n = 10) or high fat diet (HFD group, n = 20) for 10 weeks. HFD mice were then randomly divided into HFD fed only group (HFD group, n = 10) and HFD plus intraperitoneally injected Cori (20 mg/kg/day, interval for 48 h) group (HFD+Cori group), and maintained for another 8 weeks. The mice fed with NCD were add to serve as a control. **(A)** Chemical structure of Cori, and CAS number :23094-69-1. **(B)** Liver gross morphology, liver sections by H&E staining, hepatosteatosis by Oil-red O(ORO)staining (Scale bar = 20 μm). **(C)** Nonalcoholic fatty liver disease (NAFLD) activity was scored based on steatosis score, inflammation score and ballooning score. **(D)** Liver weights of each group. **(E)** Liver index was calculated as the ratio of liver weight to body weight (%). **(F)** Epididymal fat index was calculated as the ratio of epididymal fat to body weight (%). **(G)** Positive area of ORO stained section (%). **(H, I)** Hepatic triglycerides (TG) and cholesterol (TC) content in the liver homogenates of each group. **(J, K)** Biochemical analysis of alanine aminotransferase (ALT) and aspartate aminotransferase (AST). **(L–N)** Real-time PCR (RT-PCR) analysis of lipogenic genes (FASN, ACC1, and SREBP-1c) and genes involved in β-oxidation of fatty acids (PPARα, CPT1α, ACOX1) and genes related to proinflammatory cytokines (MCP1, F4/80, TNF-α, IL-6). All data are presented as means ± SD (n = 10 mice/group). ^#^*p* < 0.05, ^##^*p* < 0.01, ^###^*p* < 0.001 vs. NCD group; *p < 0.05, **p < 0.01, ***p < 0.001 vs. HFD group.

Corilagin (Cori, **Figure 1A**), a polyphenols tannic acid compound, has been found in many ethnopharmacological plants, such as Phyllanthus reticulatus, Geranium wilfordii, Phyllanthus emblica, and Dimocarpus longana. Originally, people were unaware of its bioactivity. Cori was firstly reported to display the pharmacological activity of antitumor by blocking the reverse transcriptase of RNA tumor viruses (Kakiuchi et al., 1985). Recently, Cori has been reported to display promising pharmacological properties, including antiinflammatory (Zhao et al., 2008), antioxidant (Wu et al., 2010), antitumor (Ming et al., 2013), as well as hepatoprotective effect. Particularly, the hepatoprotective effect of Cori has been brought into focus by current research. Several studies reported the protective role of Cori against hepatocellular carcinoma (HCC) (Ming et al., 2013), hepatic injury following hemorrhagic shock (Liu et al., 2017), schistosomiasis hepatic fibrosis (Li et al., 2017), and hepatitis c virus (HCV) infection (Reddy et al., 2018). However, whether Cori possesses the therapeutic potential for the treatment of NAFLD has not been reported yet.

In this study, we investigated the beneficial effects of Cori on improving NAFLD and explored the possible mechanism. Our results demonstrated that Cori ameliorated NAFLD in HFD-induced mice and attenuated PA/OA-induced lipid accumulation in hepatocyte cell line alpha mouse liver 12 (AML12) cells. Mechanistically, Cori alleviated lipid deposition in livers of HFD-induced mice *via* diminishing oxidative stress, restoring autophagic flux, and enhancing mitochondrial function.

## Results

### Cori Alleviated Hepatic Lipid Accumulation in HFD-Induced C57BL/6 Mice

To investigate the role of Cori in the development of NAFLD associated with diet-induced obesity, the 6-week-old male C57BL/6 mice were fed with HFD for 10 weeks and were then given with or without Cori (20 mg/kg, interval for 48 h) by the intraperitoneal injection (i.p.) for another 8 weeks. The mice fed with NCD were added to serve as the control. At the end of experiments, the mice were dissected for further investigation. Compared with the NCD group, the liver gross morphology of the HFD group was obviously pale and enlarged, whereas the appearance of the livers in Cori-treated group was almost normal (**Figure 1B**). Moreover, the liver weights of HFD group were obviously heavier than those in NCD group (**Figure 1D**). However, we did not find significant difference in liver weights between HFD+Cori group and NCD group (**Figure 1D**). Liver index (**Figure 1E**) and EFP/BW ratio (**Figure 1F**) were also markedly decreased after Cori treatment compared with HFD group. Hematoxylin-eosin (H&E) staining of livers in the HFD group displayed vacuolation indicative of lipid deposition inside the liver parenchyma cells (**Figure 1B**) and Oil-red O staining of the frozen liver sections of HFD group exhibited more serious hepatocyte steatosis (**Figures 1B, G**). As shown in **Figure 1C**, NAFLD activity in HFD group was higher than that in NCD group, whereas Cori administration tended to counteract this effect. These changes of histopathology were remarkably ameliorated by treatment with Cori as evidenced by decreased lipid accumulation (**Figure 1B**).

To further investigate whether Cori has beneficial effect on whole-body and hepatic lipid profiles, we next conducted biochemical analysis. Compared with NCD group, HFD group exhibited elevated serum total TC, TG, LDL-C levels as well as hepatic TG, TC contents (**Figures 1H, I**), but did not show significant difference in serum HDL-C between the two groups (**Table 1**). However, Cori treatment significantly improved serum and hepatic lipid profiles under HFD conditions. In particular, HDL-C, a negative predictor of NAFLD, was greatly elevated in Cori treatment group relative to those of HFD group (**Table 1**). As shown in **Figures 1J, K**, the serum AST and ALT levels were both significantly increased in HFD groups compared to that of mice in NCD groups. Whereas, the levels of serum AST and ALT in HFD+Cori groups were not apparently affected after Cori treatment relative to that of mice in NCD groups. The results suggested that Cori had no harmful effects on the liver function.

**Table 1:** Plasma metabolic variables in mice of normal chow diet (NCD), high-fat diet (HFD), and HFD+Cori groups.

Parameters	NCD	HFD	HFD+cori
TG (mM)	0.90 ± 0.03	1.21 ± 0.05#	0.95 ± 0.07*
TC (mM)	2.97+0.09	4.14 ± 0.12##	3.09 ± 0.11**
LDL(mM)	0.83+0.07	1.37 ± 0.09#	0.88 ± 0.05*
HDL(mM)	1.51+0.09	1.81 ± 0.14	2.31 ± 0.12*

All data are presented as means ± SD (n = 10 mice/group). #p < 0.05, ##p < 0.01 vs. NCD group; *p < 0.05, **p < 0.01 vs. HFD group.

We next examined the expression of genes associated with lipid metabolism by RT-PCR analysis. As shown in **Figure 1L**, Cori treatment significantly downregulated the mRNA expression of genes related to fatty acid synthesis including FASN, ACC1, and SREBP-1c and remarkably upregulated the expression of genes related to fatty acid oxidation including PPARα, CPT1α, and ACOX1 (**Figure 1M**). Additionally, the mRNA expression levels of genes related to proinflammatory cytokines including MCP1, F4/80, TNF-α, and IL-6 were obviously increased in HFD group, whereas Cori treatment blocked HFD-induced inflammatory responses (**Figure 1N**). Taken together, these results indicated that Cori prevented against the pathological process of NAFLD by decreasing lipid deposition, which was involved in the role of Cori in maintaining hemostasis of lipid metabolism and inflammatory responses

### Cori Restored Defective Autophagy in HFD-Induced Fatty Liver

**Figure 2:**
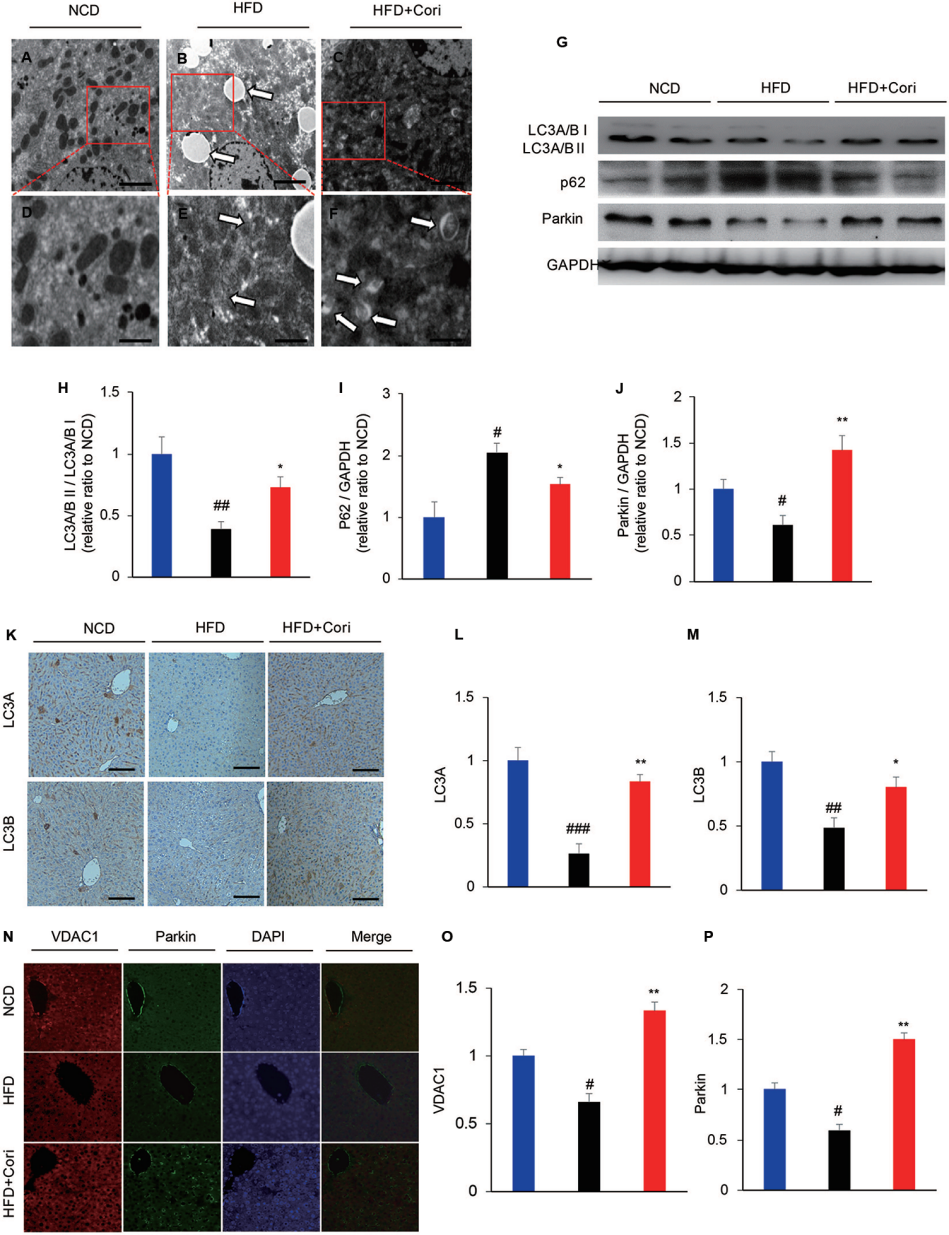
Cori ameliorated lipid deposition by enhancing autophagy levels in liver of high-fat diet (HFD)-induced C57BL6 mice. **(A–F)** Transmission electron micrographic (TEM) analysis of liver sections from normal chow diet (NCD) mice (**A**, highlighted in **D**), HFD mice (**B**, highlighted in **E**), HFD+Cori mice (**C**, highlighted in **F**). (**A–C** magnification × 5 × 103; **D–F** magnification ×2×104). The white arrows highlight lipid droplets **(B)**, deformed mitochondria **(E)** or autophagosomes **(F)**. Scale bars = 2 μm **(A–C)** and 0.5 μm **(D–F)**. **(G)** Western blot and **(H–J)** semiquantitative analysis of autophagy related protein LC3A/B I, LC3A/B II, p62, Parkin in livers of each group. GAPDH was detected as a loading control **(H–J)**. **(K)** Immunohistochemistry and **(L, M)** quantitative analysis of LC3A, LC3B in livers of each group. Images of cells were visualized by fluorescent microscope (magnification ×200). **(N)** Immunofluorescence and **(O, P)** quantitative analysis of Parkin (green) and the mitochondrial marker VDAC1 (red) in liver of mice (yellow/orange positive fields represented Parkin/VDAC1 double labeled mitochondria). Images of cells were visualized by fluorescent microscope (magnification ×400). All data are presented as means ± SD (n = 10 mice/group). ^#^*p* < 0.05, ^##^*p* < 0.01, ^###^*p* < 0.001 vs. NCD group; *p < 0.05, **p < 0.01 vs. HFD group.

It has been shown that defective autophagy contributed to lipid deposition and accelerated the onset of NAFLD (Singh et al., 2009). To determine whether Cori attenuated HFD-induced liver steatosis by activating autophagy pathway, we employed transmission electron microscope (TEM) to observe presence of autophagosomes. When placed HFD, Cori treatment obviously promoted the maturation of autophagosomes in comparison with the mice fed with HFD only as observed by increased numbers of mitochondria in autophagic double vesicles (autolysosomes; fused autophagosomes and lysosomes) (**Figures 2A–F**), suggesting that Cori played a key role in the autophagy activation.

Light chain3-II (LC3-II), which is lipidation of LC3 often used as an indicator of autophagosomes induction (Tanaka and Hikita, 2016). Our results showed that lower LC3A/B II protein levels and higher sequestosome-1 (SQSTM1)/p62 (a selective substrate for autophagy (Tanaka and Hikita, 2016) protein levels in the livers compared with NCD group (**Figure 2G**). Cori treatment restored HFD-induced decline in autophagy activity, which was supported by markedly increased protein expression of LC3A/B II and decreased p62 levels under HFD conditions (**Figures 2G–J**). Furthermore, Immunohistochemistry analysis demonstrated LC3 protein levels were significantly increased in livers of Cori-treated mice compared with HFD only group (**Figures 2K–M**).

Mitophagy is a selective autophagic process of mitochondria that removes damaged or unwanted mitochondria (Sinha et al., 2015). Parkin, an E3 ubiquitin ligase, mediates mitophagy through selectively recruitment to the impaired mitochondria that are subsequently engulfed and degraded (Narendra et al., 2008). Our results showed that Parkin protein levels were significantly increased in the livers of Cori+HFD mice compared with HFD only mice by western blot (**Figures 2N–P**), emphasizing that Cori prevented hepatic steatosis partly *via* enhancing Parkin-mediated mitophagy. The results were further confirmed by colocalization between Parkin and voltage-dependent anion channel 1 (VDAC1; a major component of the outer mitochondrial membrane) using immunofluorescence staining. Our results showed there was little colocalization of Parkin and VDAC1 in HFD group, whereas Cori enhanced the colocalization between Parkin and VDAC1 (**Figure 2I**). Taken together, these findings indicated that Cori improved lipid deposition in the livers of HFD mice, which was associated with enhanced activity of autophagy, including mitophagy.

### Cori Prevented From PA/OA-Induced Lipid Deposition in AML12 Cells

**Figure 3:**
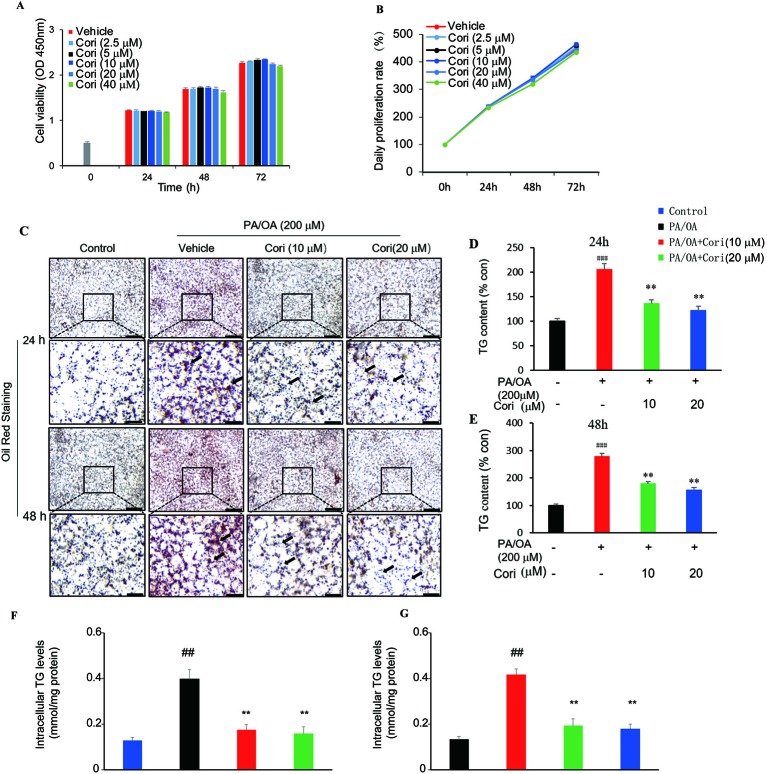
Cori attenuated PA plus OA-induced intracellular lipid deposition in AML12 cells. **(A, B)** AML12 cells were treated with different concentrations of Cori (0, 2.5, 5, 10, 20, 40 μg/ml) for 24, 48, and 72 h, respectively. The relative cell viability was measured by CCK-8 assay. **(C)** AML12 cells were exposed to a mixture of 200 μM PA/OA in the presence or absence of Cori (10, 20 μM) for 24, 48h, respectively. Lipid droplets accumulation within the AML12 cells were determined by Oil-red O staining. AML12 cells cultured in standard medium were add to serve as the control. **(D–G)** Intracellular triglycerides (TG) contents were quantified by its optical density (OD) value at 540 nm **(D, E)** and by Triglyceride assay kit **(F, G)**. Data were presented as mean ± SD of three independent experiments; each performed in triplicates. ^###^*p* < 0.001, ^##^*p* < 0.01 vs. control group; **p < 0.01 vs. PA/OA only group **(F, E)**.

Next, we examined the effect of Cori on lipid accumulation in AML12 liver cells. Prior to testing the effect of Cori in AML12 cells, CCK-8 assay was performed to evaluate the cytotoxic effects of Cori on AML12 cells. The cells were treated with Cori at various concentrations (2.5, 5, 10, 20, 40 μM) and for different time periods (24, 48, and 72 h). Our results showed that AML12 cells treated with tested concentrations of Cori did not exhibit obviously cytotoxic effect on cell growth and viability (**Figures 3A, B**). Based on these results, 10 μM and 20 μM concentrations of Cori were used as low-dose and high-dose treatment for the following *in vitro* experiments.

**Figure 4:**
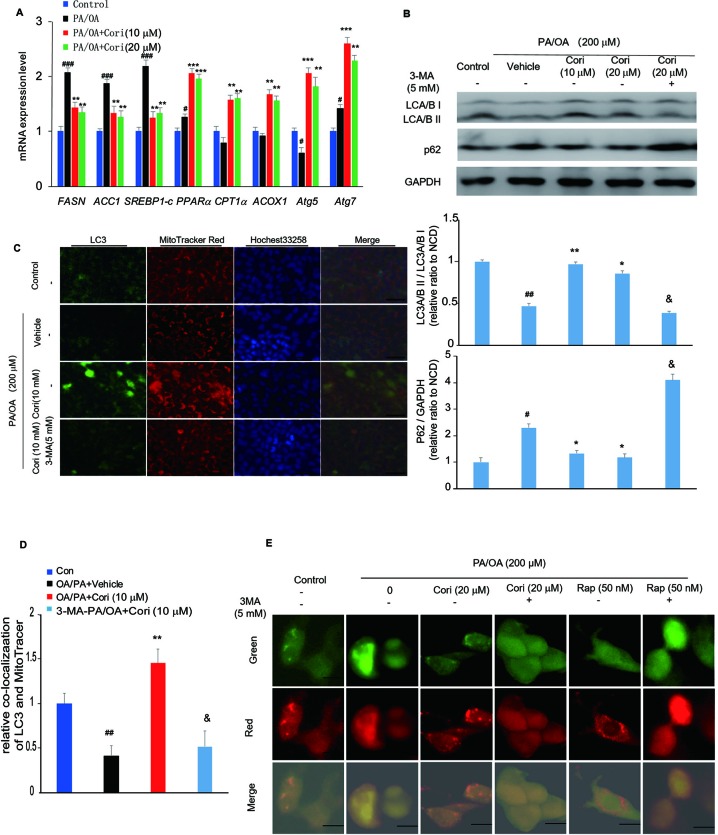
Cori ameliorated nonalcoholic fatty liver disease (NAFLD) by restoring autophagic flux *in vitro*. **(A)** AML12 cells were treated with 200 μM PA/OA in the absence or presence of Cori (10 or 20 μM) for 24 h. The relative expression levels of lipogenic genes (SREBP-1c, ACC1, and FASN), gene involved in β-oxidation of fatty acids (CPT1α, PPARα, ACOX1), and autophagy-related protein (Atg7, Atg5) were determined by RT-PCR assay. **(B)** Cells were exposed to 200 μM PA/OA and in the presence or absence of Cori (10 or 20 μM) for 24 h. 3-MA (5 mM) were add to serve as autophagic inhibitor. Western blot and semiquantitative analysis of autophagy related protein LC3A/B I, LC3A/B II, p62 in hepatocytes of each group. GAPDH was detected as a loading control. **(C)** Immunofluorescence stained with antibodies against LC3 (green) and the mitochondrial marker MitoTracker (red) in AML12 cells. **(D)** Quantitative analysis of yellow/orange positive fields represented LC3/MitoTracker double labeled mitochondria from **(C)**. Images of cells were visualized by fluorescent microscope. **(E)** Cells were mixed with Ad-mCherryGFP-LC3B and then treated with Cori for 24 h, the mCherry (red) and GFP (green) were analyzed by fluorescence microscopy (Scale bar = 20 μm). Data were presented as mean ± SD of three independent experiments; ^#^*p* < 0.05, ^##^*p* < 0.01, ^###^*p* < 0.001 vs. NCD group; *p < 0.05, **p < 0.01, ***p < 0.001 vs. high-fat diet (HFD) group. ^&^*p* < 0.05 vs. HFD group.

PA/OA have been widely used to establish models of NAFLD *in vitro* (Ziamajidi et al., 2013; Cai et al., 2014). In this study, AML12 cells were treated with PA/OA (200 μM) and the tested concentrations of Cori (10 μM or 20 μM) for 24 and 48 h, respectively. Our results showed that PA/OA-treated AML12 cells exhibited more obvious intracellular lipid accumulation compared with control cells (without PA/OA treatment), as evidenced by the increased number and size of lipid droplets (highlighted by arrows in **Figure 3C**) using Oil-red O staining, indicating that we successfully established a cell culture model of PA/OA-induced hepatic steatosis (**Figure 3C**). However, Cori treatment clearly alleviated PA/OA-induced lipids accumulation (**Figure 3C**). Furthermore, intracellular TG contents were measured to quantitatively analyze the extent of lipid accumulation. Compared with AML12 cells treated with PA/OA only (vehicle treatment), the intracellular TG contents were obviously decreased by combination of treatment with PA/OA+Cori, though the extent of decreased lipid droplet was no significance after Cori treatment with low dose(10 μM) and high dose(20 μM) (**Figures 3D–G**). In addition, Cori treatment also restored the expression of genes related to lipid metabolism in accordance with our results *in vivo* (**Figure 4A**). Taken together, these findings indicated that Cori prevented against PA/OA induced hepatic steatosis in a cell-autonomous way.

### Cori Restored Autophagic Flux in PA/OA Treated AML12 Cells

To determine whether the amelioration of hepatic lipid deposition by Cori treatment *via* autophagy activation, we next examined the protein expression levels of LC3 and p62 in our established model of NAFLD *in vitro*. Compared with control cells, PA/OA- treated cells exhibited impaired autophagy activity as evidenced by decreased LC3A/B II protein levels and increased p62 protein levels by western blot analysis. However, Cori substantially increased LC3A/B II levels and decreased p62 levels (**Figure 4B**), indicating that Cori possessed potential to reverse PA/OA-induced impairment of autophagy activity. Importantly, 3-Methyladenine (3-MA, an autophagy inhibitor) treatment increased p62 protein levels in AML12 cells (**Figure 4B**), suggesting that Cori played a key role in autophagy activation by restoring autophagic flux. Likewise, mRNA expression levels of certain autophagic pathway factors involved in autophagosomes formation, Atg7 and Atg5 (Tanaka and Hikita, 2016), were increased in Cori-treated cells by RT-PCR assay (**Figure 4A**), further confirming that Cori enhanced autophagic flux *in vitro*.

Given LC3 is the marker of autophagosomes, we performed Immunofluorescence to visualize the progression of autophagosomes in PA/OA-treated AML12 cells. As shown in **Figure 4C**, the cellular expression levels of LC3 were reflected by the intensity of green ﬂuorescence. The intensity of green ﬂuorescence was markedly decreased in AML12 cells exposed to PA/OA, whereas Cori treatment significantly increased the intensity of green ﬂuorescence (**Figure 4C**). Importantly, treatment with Cori obviously enhanced colocalization of LC3 and a mitochondrial marker (MitoTracker Red) as evidenced by the enhanced intensity of the merged ﬂuorescence. Furthermore, pretreatment of cells with 3-MA decreased LC3 protein levels and diminished colocalization of LC3 and mitochondria (**Figures 4C, D**). In addition, as shown in **Figure 4E**, compared with the AML12 cells treated with PA/OA only, Cori treatment increased both autophagosome (yellow puncta) and autolysosome (red puncta) formation in AML12 cells exposed to PA/OA, while Cori-mediated enhanced autophagic flux was inhibited by combination with 3-MA. In addition, we observed the similar results after Rap (autophagy activator) treatment. These results are consistent with the results in **Figure 4B** and **Figures 4C, D**, thus indicating the facilitating roles of Cori in autophagy initiation and flux. Taken together, these results indicated that Cori can attenuate the decrease of autophagy flux in AML12 cells after exposure to PA/OA.

### Cori Suppressed Intracellular PA/OA Induced ROS Levels

**Figure 5:**
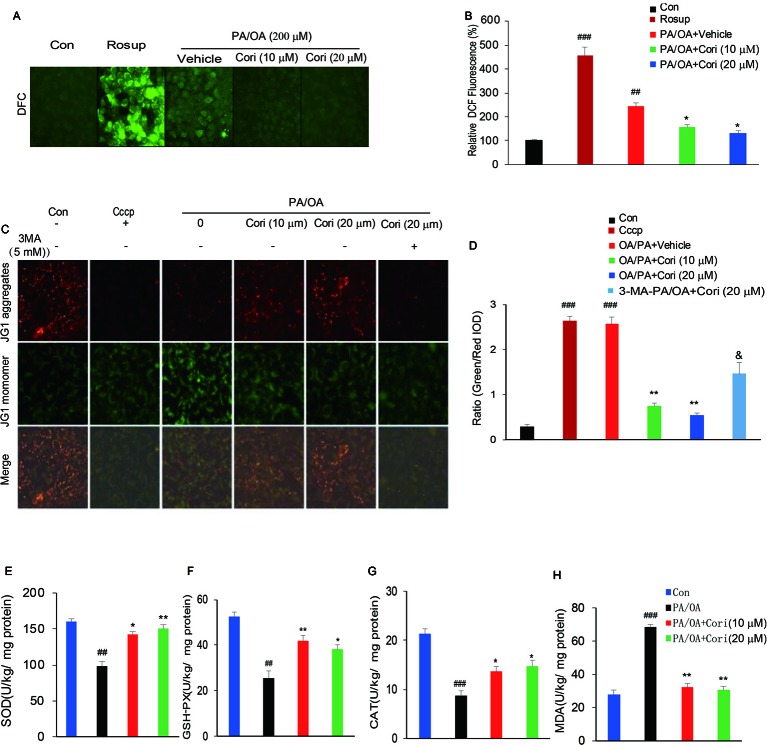
Cori protected against PA/OA induced reactive oxygen species (ROS) overproduction, decline in mitochondrial membrane potential as well as antioxidative defense system. **(A, B)** AML12 cells were exposed to 200 μM PA/OA in the absence or presence of Cori (10 or 20 μM) for 24 h. ROS-sensitive fluorescent probe DCFH-DA was used to detect intracellular ROS levels. Rosup was add to serve as the positive control. Images of cells were visualized by fluorescent microscope. **(C, D)** AML12 Cells were exposed to 200 μM PA/OA in the absence or presence of Cori (10 or 20 μM) for 24 h. JC-1 was used to monitor mitochondrial membrane potential in cells. Autophagic inducer Cccp (10 μM) or autophagic inhibitor 3-MA (5 mM) was add to serve as the positive or negative control, respectively. Images of cells were visualized by fluorescent microscope. **(E–H)** AML12 Cells were exposed to 200 μM PA/OA in the absence or presence of Cori (10 or 20 μM) for 24 h. The effects of Cori on SOD **(E)**, GSH-Px **(F)**, CAT **(G)**, and malondialdehyde (MDA) **(H)** levels were determined by ELISA assay. The data was presented as mean ± SD of three independent experiments; each performed in sextuplica. ^##^*p* < 0.01, ^###^*p* < 0.001 vs. control group; *p < 0.05, **p < 0.01 vs. PA/OA only group. ^&^*p* < 0.05 vs. Cori (20 μM) group.

It has previously been reported that ROS overproduction promotes neutral lipid accumulation in hepatocytes, thus contributing to the development of NAFLD (Ferramosca et al., 2017; Braud et al., 2017). To determine whether Cori alleviated PA/OA-induced intracellular fat accumulation through suppressing intracellular ROS levels, we next measured the intracellular ROS levels by using DCFH-DA, a peroxide/redox-sensitive fluorescent probe. Consistent with the increased ROS levels by Rosup treatment (positive control), we found that the levels of ROS were significantly increased after exposure to PA/OA for 24 h in AML12 cells. By contrast, Cori obviously decreased the levels of ROS under PA/OA conditions, as observed by the reduction of fluorescent intensity (**Figures 5A, B**). These results indicated that Cori may reverse PA/OA-induced intracellular lipid accumulation through suppressing ROS levels.

Previous reports have demonstrated that oxidative stress resulted from ROS overproduction is a pivotal factor causing mitochondrial dysfunctions (Wang et al., 2015).To further confirm whether Cori improved PA/OA induced mitochondrial dysfunction, we next evaluated mitochondrial function by monitoring mitochondrial membrane potential (ΔΨm) in AML12 cells using JC-1 fluorescent dye. Consistent with the decreased ΔΨm levels by Cccp treatment (positive control), a marked drop in ΔΨm levels were represented after exposure to PA/OA for 24 h in AML12 cells, as evidenced by the elevated ratio of green to red fluorescence intensity. In contrast with this, PA/OA-induced decrease in ΔΨm was improved by both L- Cori (10 μM) and H- Cori (20 μM) treatment. Furthermore, Cori-mediated increase in ΔΨm was significantly inhibited by using autophagy inhibitor 3 MA (**Figures 5C, D**). These results suggested that Cori played a protective role against oxidative stress-induced mitochondrial injury, which was associated to enhanced activity of autophagy.

Excessive ROS production overwhelmed the capacity of antioxidant defense system, which further promoted oxidative stress (Shin et al., 2019). To determine whether Cori enhanced the activities of enzymes involved in antioxidant defense system, we next investigated major antioxidant enzymes levels in AML12 cells. Our results demonstrated that the activities of the antioxidative indices SOD (**Figure 5E**), GSH-Px (**Figure 5F**), as well as CAT (**Figure 5G**) were found significantly decreased in PA/OA induced hepatocytes, as compared to the control cells. MDA, a product of lipid peroxidation of phospholipids, were induced by excessive ROS, thus reflecting the levels of intracellular ROS. In this context, Cori treatment improved PA/OA-induced increase of MDA levels in hepatocytes (**Figure 5H**). Taken together, these results indicated that Cori could protect from PA/OA-induced oxidative injury in mitochondria by enhancing the activity of antioxidant defense system.

### Cori Protected Against ROS-Induced Mitochondrial Dysfunction in AML12 Cells

**Figure 6:**
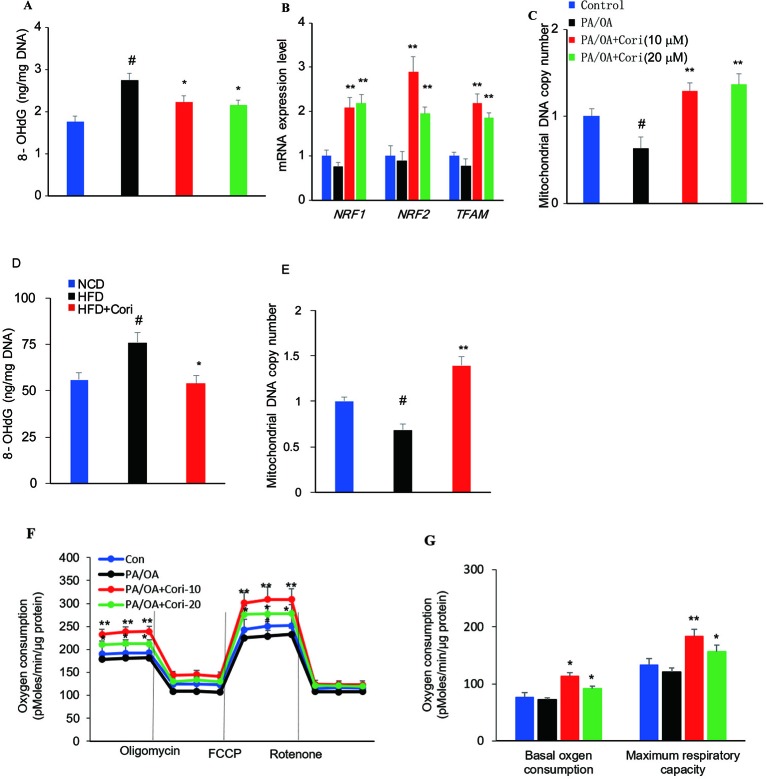
Cori protected against reactive oxygen species (ROS)-induced mitochondrial dysfunction in AML12 cells. **(A–C)** AML12 cells were exposed to 200μM PA/OA in the presence or absence of Cori (10 or 20 μM) for 24 h. The levels of oxidative DNA damage marker 8-OHdG were determined by ELISA assay **(A)**. Real-time PCR (RT-PCR) analysis of genes involved in mitochondrial biogenesis, including NRF1, NRF2, Tfam **(B)**. RT-PCR analysis of mtDNA copy number **(C)**. **(D)** The levels of oxidative DNA damage marker 8-OHdG in livers. **(E)** The levels of mitochondrial mtDNA in livers. **(F)** Mitochondrial respiration capacity was determined by Seahorse assay. **(G)** Basal OCR is [OCR with substrates - OCR with rotenone and antimycin A] and maximal respiratory capacity is [OCR with FCCP - OCR with rotenone and antimycin A]. Data were presented as mean ± SD of three independent experiments; each performed in sextuplica. ^#^*p* < 0.05 vs. control group; ^*^p < 0.05, **p < 0.01 vs. PA/OA only group.

ROS-mediated mitochondrial dysfunction can cause ineffective capacity of mitochondrial fatty acid oxidation, which inversely aggravates excessive ROS production, thus constituting a vicious cycle in the progression of NAFLD (Wang et al., 2015). To evaluate the degrees of mitochondrial DNA damage caused by ROS, we next measured 8-OHdG (a marker of oxidative DNA damage) levels in hepatocytes (Du et al., 2017). In comparison with control cells, AML-12 hepatocytes exposed to PA/OA exhibited increased 8-OHdG levels, while Cori treatment alleviated the mitochondrial DNA damage due to PA/OA treatment (**Figure 6A**).

To further clarify the regulatory role of Cori on mitochondrial biogenesis, RT-PCR was performed to analyze the expression of mitochondrial biogenesis related gene NRF1, NRF2, and TFAM. Our results demonstrated that the expression levels of NRF1, NRF2, and TFAM were significantly downregulated. However, the expression levels of these genes can be upregulated after Cori treatment (**Figure 6B**), indicating Cori was responsible for manipulating the expression of several key genes involved in mitochondrial biogenesis. Consistent with PA/OA-mediated exacerbation of mtDNA damage, we found remarkable reduction of mtDNA copy number in PA/OA-treated cells. Interestingly, Cori treatment exhibited a significant increase in mtDNA copy number (**Figure 6C**), suggesting that Cori improved the depletion of mtDNA content caused by PA/OA. Besides, Cori treatment also ameliorated mitochondrial dysfunction evidenced by decreasing 8-OHdG levels and increasing mtDNA copy number in livers of NAFLD models (**Figures 6D, E**).

To determine whether Cori improved the impaired mitochondrial respiratory capacity caused by ROS in PA/OA-treated cell models of hepatic steatosis, we next measured mitochondrial oxygen consumption rates (OCR) under basal and stress conditions in AML12 cells using Seahorse analysis. In our study, Cori-treated (notably at the dose of 10 μM) AML12 cells exhibited enhanced mitochondrial respiratory capacity as compared to cells exposed to PA/OA only, especially in basal oxygen consumption as well as maximum respiratory capacity (**Figures 6F, G**). Taken together, these results indicated that Cori ameliorated ROS-induced mtDNA damage, as well as enhanced the mitochondrial activity in AML12 cells.

## Discussion

The excessive lipid accumulation is the hallmarker of NAFLD, displaying in more than 5% of hepatic steatosis that occupy 5%–10% of liver weight (Sanyal et al., 2011). Mitochondria are the main site involved in free fatty acid (FFA) metabolism. A growing body of evidence suggests that NAFLD might be a mitochondrial disease (Jiang et al., 2013; Peng et al., 2018). Lipid-overloaded liver initiates mitochondrial adaptions on FFA metabolism. However, these adaptations are not sufficient to curb fat accumulation in livers and secondarily induced ROS overproduction in this process due to the enhanced mitochondrial fatty acid oxidation (mtFAO) (Begriche et al., 2013). On the other hand, overproduction of ROS, in turn, impairs mitochondrial dysfunction, which could aggravate NAFLD (Oliveira et al., 2002; Lee et al., 2019). Similarly, although ROS has been shown to play an essential role in the initiation of autophagy (Sinha et al., 2015), it blocks autophagic flux due to decompensated mitochondrial adaptations, which is also considered as an important factor accelerating the physiopathology of NAFLD (Jiang et al., 2013). In this study, we focused on a previously unreported drug-Cori, illuminating that Cori treatment could ameliorate NAFLD in HFD-induced mice and attenuate PA/OA-induced lipid accumulation in AML12 cell *via* diminishing oxidative stress, restoring autophagic flux, and enhancing mitochondrial function.

We firstly established the murine model of NAFLD by feeding mice with HFD for 10 weeks. We observed a decline in lipid droplets accumulation of livers of HFD-fed mice (**Figure 1B**) or PA/OA induced hepatocytes (**Figure 3C**) after treatment with Cori. Besides, Cori also possessed the beneficial effect on ameliorating the serum lipid profiles and hepatic TG, TC contents compared with those in HFD-fed only mice (see **Table 1**). Our data showed that Cori significantly inhibited the expression of genes involved in lipogenesis, including FASN, ACC1, and SREBP1-c (**Figure 1L**). However, Cori also enhanced fatty acids β-oxidation associated genes (PPARα, CPT1α, ACOX1) (**Figure 1M**), which suggested mitochondrial function was enhanced by Cori treatment. In summary, the present results suggested Cori ameliorated lipid metabolism disorders in hepatocytes, at least in part, through reducing the expression of genes related to lipogenesis and restoring genes involved in fatty acids β-oxidation of mitochondria at transcriptional levels.

Next, we investigated whether autophagy is associated with Cori-mediated reduction of lipid deposition *in vivo* and *in vitro*. Autophagy is quite essential for maintaining the balance of lipid metabolism in hepatocytes (Singh et al., 2009). However, excessive nutrients supply for ATP production can induce autophagy inhibition (Czaja et al., 2013). Several reports have revealed that defective autophagy led to increased hepatic TG and lipid droplets (LDs), thus contributing to the progression of NAFLD (Singh et al., 2009; Wang et al., 2015). In our study, we found that Cori treatment significantly increased LC3A/BII protein levels and decreased p62 protein levels under HFD conditions (**Figure 2G**). Previous studies have investigated the effects of FFAs on autophagic flux using human nontumor hepatic cell line L02 cells (human hepatic L02 cells) and found that autophagic flux was blocked at the late hour (24,36 h) of steatosis due to homeostatic imbalance (Liu et al., 2018; Kim et al., 2018; Zhang et al., 2019).Likewise, our results were further confirmed *in vitro* experiments (**Figure 4B**). In particular, combined treatment with autophagy inhibitor (3-MA) evidently inhibited Cori-mediated decrease of p62 protein levels (**Figure 4B**). Collectively, these results indicated that Cori have the potential to prevent HFD-mediated autophagy impairment by restoring autophagic flux.

Mitophagy is a process that selectively degradates damaged mitochondria, which is essential for quality control of mitochondria through eliminating aged or damaged mitochondria and renewing organelles (Wang et al., 2015). Furthermore, our results showed that Cori significantly enhanced Parkin-mediated mitophagy, as evidenced by significantly increased Parkin protein levels in the livers of Cori-treated mice under HFD (**Figure 2G**). For further verification, we found that Cori-treated mice exhibited the enhanced colocalization between Parkin and VDAC1 using immunofluorescence staining (**Figures 2N–P**). Consistent with our results *in vivo*, we also found an increase in colocalization of LC3II and MitoTracker in PA/OA-induced AML12 cells after Cori treatment (**Figures 4C, D**). Moreover, compared with the AML12 cells treated with PA/OA only, Cori treatment increased both autophagosome (yellow puncta) and autolysosome (red puncta) formation in AML12 cells (**Figure 4E**). Collectively, our results suggested that Cori alleviated hepatic steatosis by activation of the autophagy pathway, especially by mitophagy-mediated signaling pathway.

Defective autophagy is closely associated with mitochondrial oxidative stress (Scherz-Shouval and Elazar, 2011), which reflects an imbalance between availability of ROS and the cellular antioxidant system (Shin et al., 2019). Next, we investigated whether Cori played a key role in reducing ROS levels in PA/OA treated cells models of NAFLD. Our results showed that intracellular ROS levels were remarkably increased in PA/OA-treated AML12 cells. However, intracellular ROS levels were significantly decreased after Cori treatment (**Figure 5A**). Recent reports have revealed that increased proinflammatory cytokines levels were a pivotal factor triggering ROS formation as well as inducing oxidative stress, which was related to HFD-induced lipid droplet accumulation (Du et al., 2017). Our results also showed that Cori treatment significantly decreased the expression of proinflammatory cytokines in livers of HFD-fed mice, including TNF-a, IL-6 (**Figure 1N**). These results suggested that Cori reduced ROS levels, which was likely associated to its antiinflammatory effects. Given that mitochondria is the major target organelle of ROS production, increased ROS levels may impair mitochondrial function, including the reduction of mitochondrial membrane potential (ΔΨm) and the onset of mitochondrial permeability transition (MPT), both of which are characteristic features of dysfunctional mitochondria (Wang et al., 2015). As such, we next detected the ΔΨm with or without Cori treatment in PA/OA-induced AML12 cells. As our expected, Cori significantly inhibited PA/OA-induced decrease of ΔΨm, whereas the effect was totally abolished by combined treatment with autophagy inhibitor (3-MA). Collectively, these results indicated that Cori prevented mitochondrial oxidative injury which induced by ROS overproduction.

Reduction of antioxidant defense activity is also a key factor triggering oxidative stress (Li et al., 2016). Previous studies have reported the decrease of antioxidant activity in obese or overweight patients of NAFLD (Krzystek-Korpacka et al., 2013). Supplement with antioxidant may attenuate ROS-induced mitochondrial dysfunction of ob/ob mice (Shin et al., 2019). Of note, Cori has been reported to exhibit strong antioxidant activity for acute liver failure (Lv et al., 2019). In our study, we found that Cori treatment restored the decreased activities of major antioxidant enzymes SOD, GSH, and CAT in PA/OA treated AML12 cells (**Figures 5E–G**). Consistent with this, the MDA (a by-product of lipid peroxidation) levels were significantly decreased after Cori treatment compared with vehicle treatment (**Figure 5H**). Collectively, these results indicated that Cori improved the oxidative injury through enhancing the activities of antioxidant enzymes to inhibit ROS overproduction in PA/OA induced AML12 cells.

When oxidative injury continuously attacked mitochondrial organelles, it can contribute to mitochondrial fatigue and eventually causes mitochondrial dysfunction, which have been observed prior to the natural history of NAFLD in obese, Otsuka Long-Evans Tokushima Fatty (OLETF) rat (Rector et al., 2010). mtDNA that encodes mitochondrial respiratory chain (MRC) polypeptides, presented in several copies within mitochondrial matrix was susceptible to oxidative damage (Xiao et al., 2017). In our study, we found that Cori treatment significantly decreased 8-OHdG levels and increased mtDNA copy number *in vivo* (**Figures 6D**, **E**) and *in vitro* (**Figures 6A–C**) NAFLD models, suggesting that Cori prevented ROS-mediated mtDNA damage. Furthermore, we also found that Cori treatment significantly increased the expression of genes related to mitochondrial biogenesis, including NRF1, NRF2, and TFAM (**Figure 6B**). These results indicated that Cori promoted the mitochondrial metabolism and organelle biogenesis. Mitochondrial β-oxidation is a key indicator of mitochondrial function (Wang et al., 2015). Consistent with the unregulated gene expression *in vitro*, we found remarkably increased mitochondrial respiratory in PA/OA-treated AML12 cells compared with vehicle treatment (**Figures 6F, G**), further demonstrating an enhanced mitochondrial function by Cori treatment. Collectively, Cori improved mitochondrial function during the process of oxidative injury, which was involved in mitigating the mtDNA damage, increasing mitochondrial biogenesis, and enhancing mitochondrial respiratory capacity.

**Figure 7:**
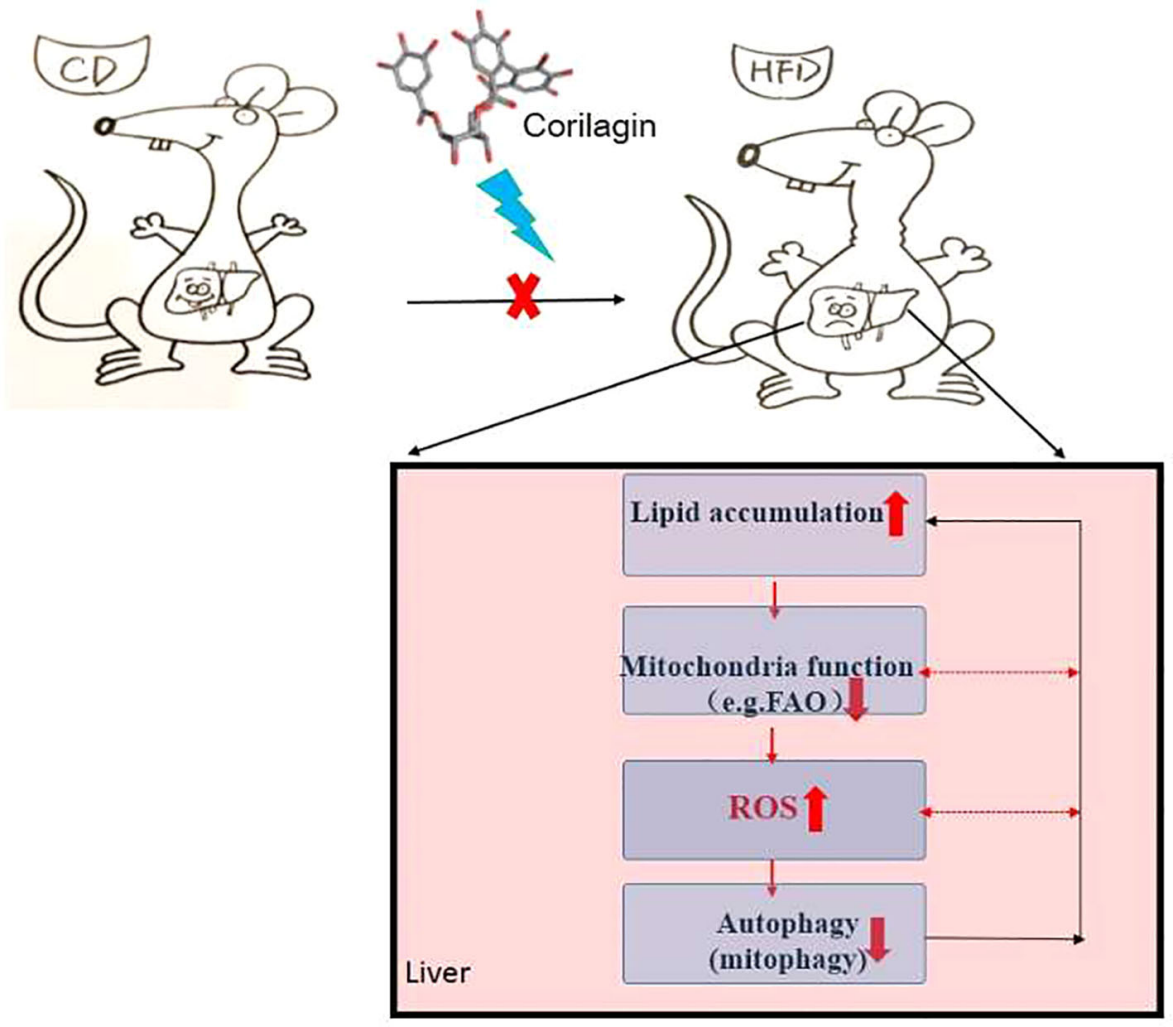
A self-perpetuating vicious cycle in the development and progression of nonalcoholic fatty liver disease (NAFLD). In the case of hypernutrition, mitochondrial adaptations (e.g., increased mitochondrial fatty acid oxidation, mtFAO) are initiated to curb fat accumulation in livers. However, this adaption secondarily induces excessive reactive oxygen species (ROS) production, thus leading to the aggravation of mitochondrial dysfunction. Underlying mitochondrial dysfunction could further accelerate ROS overproduction and the progression of NAFLD. On the other hand, ROS potentiates the dual role in the initiation of autophagy and suppression of autophagic flux. Overproduction of ROS continuously attacks mitochondria, causing mitochondrial fatigue and mitochondrial decompensation, which promotes lipid overload and lipotoxicity and eventually blocks the autophagic flux. Meanwhile, defective autophagy alters lipid homeostasis, which contributes to the lipid accumulation in liver. In this study, the Chinese herb, Corilagin (Cori) exerts the effects on alleviating lipid deposition in livers of high-fat diet (HFD) induced C57BL/6 mice *via* diminishing oxidative stress, restoring autophagic flux arrest, as well as improving mitochondrial functions.

In conclusion, there is “a self-perpetuating vicious cycle” that involved in the interactions among autophagy, mitochondrial dysfunction and ROS overproduction, thereby contributing to the onset of NAFLD (detailed in **Figure 7**). In this study, Cori ameliorated NAFLD in HFD-induced mice and attenuated PA/OA-induced lipid accumulation in AML12 cells. Mechanistically, Cori acts on this cycle and exerted the effect on alleviating lipid deposition *via* diminishing oxidative stress, restoring autophagic flux, as well as improving mitochondrial function (**Figure 7**). Thereby, Cori should be a potential candidate for the treatment of NAFLD.

## Materials and Methods

### Cell Culture

Bulleted lists look like this: AML12 hepatocytes were obtained from ATCC (Manassas, USA) and were cultured in DMEM/F-12 medium (Life Technologies) supplemented with 10% fetal bovine serum (Life Technologies), 5 μg/ml ITS (Life Technologies) containing insulin, transferrin and selenium, 40 ng/ml dexamethasone, 100 U/ml of penicillin, 100 µg/ml of streptomycin and 0.25 g/L of glutamine. The cells were incubated at 37°C under the condition of 95% air and 5% CO_2_.

### Cells Viability Assay

Cori (CAS: 23094-69-1) was purchased from BioPurify Phytochemicals Ltd. (Purity: 98%, Catalogue No.: BP0393, Chengdu, China). Cori was first dissolved in dimethyl sulfoxide (DMSO) and stored in the dark at −20°C. During our experiments, Cori was diluted into corresponding concentration. Cells viability in response to Cori was measured by using Cell Counting Kit-8 (CCK-8; MCE, Monmouth Junction, NJ) according to manufacturer’s instructions. In brief, AML12 cells were seeded into 96-well plates (1×10^4^ cells each well), cultured for 24 h and then incubated with different concentrations of Cori (2.5, 5, 10, 20 or 40 μM) for 24, 48, or 72 h, respectively. DMSO (0.1%, v/v) treated cells served as the control, then we replaced the old medium with fresh medium containing 10% CCK-8 solution in each well and incubated for another 2 h. The daily percentage of cell viability was calculated according to the following formula: proliferation rate = (OD value of the cells (24, 48, or 72 h) – OD value of blank well (24, 48, or 72 h) /[OD value of the cells (0 h) − OD value of blank well (0 h)] × 100%. The optical density (OD) was measured at 450 nm in a microplate reader (Thermo Fisher, USA).

### Oil-Red O Staining

AML12 cells were collected and washed twice with PBS buffer. Cells were then fixed with 4% paraformaldehyde for 30 min, and then washed three times with PBS buffer. The 5% Oil-red O storage solution (Sigma Chemical, St. Louis, MO, USA) was mixed with ddH_2_O at a ratio of 3:2 and filtered to obtain the working solution. Cells were stained with Oil-red O working solution for 30 min at room temperature. Oil-red O contained in lipid droplets were extracted with isopropanol, and OD value was measured at 540 nm to quantitatively reflect the content of intracellular lipid. Intracellular triglycerides were assayed according to the method in 4.10.

### Animals and Treatments

Six-week-old male C57BL6 mice (n = 30) weighing 20 ± 2 g were purchased from the Shanghai Center of Experimental Animals (Shanghai, China; certificate number SCXK2007-0005) and mice were housed under a standard environment (20 ± 2˚C; 50 ± 5% humidity; 12:12-h light/dark cycle, diet and filtered water ad libitum). Following 1-week acclimatization period, mice were randomly divided into three groups (n = 10, per group): (i) mice fed with normal control diet for 18 weeks, (n = 10, NCD group); (ii) mice fed with high fat diet containing 60% kj fat for 18 weeks, (n = 10, HFD group); (iii) mice fed with high fat diet for 10 weeks, then feeding them high fat diet simultaneously with intraperitoneal injection Cori (20 mg/kg/day, interval for 48 h) for another 8 weeks, (n = 10, HFD+Cori group). Food intake and body weight were recorded weekly. At the end of experiment process, mice were sacrificed under deeply anesthetized to collect blood samples and liver tissues were excised and weighed for the follow-up experiments. Liver index was calculated as the ratio of liver weight to body weight. Epididymal fat index was calculated as the ratio of epididymal fat to body weight. All the experiments were approved by the ethics committee of the Ninth People’s Hospital, Medical School of Shanghai Jiaotong University, Shanghai, China (project no. SH9H-2016-TK356-1) and conducted according to the guidelines for the Care and Use of Laboratory Animals.

### Histological Examination

H&E staining was used to observe pathological changes of liver tissues. Fresh liver mass were fixed in 4% paraformaldehyde and embedded with paraffin. The liver tissues were prepared into thin slices (4-µm thickness) and these sections were stained with H&E staining according to the established methods (Xiao et al., 2017). NAFLD activity score system of H&E staining, which is based on hepatic steatosis, inflammation, ballooning as previously described (Kleiner et al., 2005). Hepatic neutral lipid deposition was evaluated by performing Oil-red O staining. The frozen liver slides (6-µm thickness) were stained with Oil-red O working solution for 8–10 min and nucleus were stained with Mayer’s hematoxylin for 30 s. After washing thoroughly, the slides were mounted with glycerin jelly for further analysis. Images were captured using the Leica MZ10 F modular stereomicroscope (Leica).

### Immunohistochemical Analysis

The embedded liver tissue was dewaxed with dimethylbenzene (I and II) and rehydrated with gradient alcohol (100%, 95%, 80%, and 70%). After performing antigen retrieval, the slices were treated with 3% H_2_O_2_ to block the endogenous peroxidase. The nonspecific binding sites then were blocked by incubating with 5% goat serum for 30 min at room temperature, and incubated with primary antibody (LC3A at 1:6400 dilution, CST; LC3B at 1:3200 dilution, CST) for overnight at 4°C. The slides were incubated with diluted HRP-labeled goat antirabbit IgG (H+L) secondary antibody at 37°C for 30 min. After washing with PBS buffer, the slides were stained with DAB and then counterstained with haematoxylin. After washing with flowing water, the sections were dehydrated. Finally, the sections were sealed with neutral balsam and covered with coverslips. The brown staining was considered to be antibody-positive.

### Immunofluorescence

For immunofluorescence analysis, liver samples or AML12 cells were processed and images were obtained as previously described (Liu et al., 2018). In brief, samples were fixed with 4% paraformaldehyde, immersed with 0.2% Triton X-100 and blocked with 10% goat albumin. They were then incubated with primary antibody(VDAC1 at 1:500 dilution, Proteintech), (Parkin at 1:300 dilution, Abcam, ab15494), LC3B (at 1:200 dilution, CST), followed by incubation with IgG-FITC-conjugated second antibody (at 1:500 dilution, Beyotime technology). Nucleus were stained with 2.5 g/ml DAPI (Sigma-Aldrich).To detect the colocalization of LC3 and a mitochondria marker (MitoTracker Red, Thermo Scientific.USA) in AML12 cells, mitochondria were prestained with MitoTracker Red (50 nM) for 30 min. Besides, we used Ad-mCherry-GFP-LC3B to monitor autophagy flux. In brief, AML12 cells were seeded (1 105 cells/well) in a 24-well-plate and infected with Ad-mCherry-GFP-LC3B (40 MOI) for 24 h and then cultured in fresh medium for another 24 h. Afterwards, the cells were treated with or without PA/OA (200 μM), Corilagin (20 μM), 3-MA (5 mM), and rapamycin (50 nM). Images were captured using the Leica MZ10 F modular stereomicroscope (Leica).

### TEM Analysis

We used TEM to observe mitochondrial ultrastructure of liver tissues in three experimental groups. Liver tissues were sliced less than 1 mm^3^ thickness, the sections were pre-fixed in 2.5% glutaraldehyde at 4°C for 2 h. After washing with sodium cacodylate buffer, tissues were postfixed with 1% osmium tetroxide solution at 4°C for 1 h. The slices were dehydrated in graded concentrations of ethanol, infiltrated with propylene oxide and embedded. The slices were sheered into 60 nm sections. Imaging was captured with transmission electron microscope (Olympus EM208S, Japan).

### Biochemical Analysis

For serum lipid profile analysis, the collected blood samples from the mice were stood at room temperature for 30 min, then centrifugation at 3,000 rpm/min for 10 min, serum levels of cholesterol (TC), triglycerides (TG), high-density lipoprotein (HDL) and low-density lipoprotein-c (LDL), aspartate aminotransferase (AST), and alanine aminotransferase (ALT) were examined using an automatic chemistry analyzer (Hitachi Ltd., Tokyo, Japan).

### Hepatic TG and TC Measurement

Hepatic TG and TC contents were assayed by using a Triglyceride assay kit (GPO-POD; Applygen Technologies Inc., Beijing, China) and Micro Total Cholestenone (TC) Content Assay Kit (Solarbio LIFE SCIENCE Co. Ltd, Beijing, China) respectively, according to the manufacturer’s instructions.

### Determination of Cellular CAT, GSH-Px, SOD, MDA Content

The assay for superoxide dismutase (SOD), glutathione peroxidase (GSH-Px), catalase (CAT), and malondialdehyde (MDA) was carried out by using commercial assay kits (Beyotime Biotechnology Co. Ltd, Shanghai, China). All the steps were taken in strict accordance with the manufacturer’s instructions (Li et al., 2016).

### mtDNA Content Quantification

Genomic DNA in AML12 cells and liver tissues was extracted using TIANamp Genomic DNA Kit (Tiangen, Biotech CO.LTD, Beijing) according to the instructions and used as a template for amplifying the mtDNA-encoded gene COXII and nuclear-encoded gene β-actin (Yuan et al., 2017). The results were shown as abundance ratio of mtDNA target gene COXII to β-actin, which correspondingly represented mitochondrial DNA and genomic DNA, respectively.

### mtDNA Damage Analysis

The extracted mtDNA was digested with DNAse I and alkaline phosphatase (TaKaRa, Otsu, Japan).Then, the levels of 8-hydroxydeoxyguanosine (8-OHdG) in hepatocytes and liver tissues were measured by a commercial ELISA kit (Cayman Chemical) according to the standard protocol (Du et al., 2017).

### Intracellular ROS Measurement

AML12 cells were seeded in 12-well plates and cultured for 24 h. Cells were stimulated with PA/OA (200 μM) in the absence or presence of tested concentration of Cori (10, 20 μM) for 24 h. The culture medium was removed, washed three times with PBS buffer and then incubated with 10 μM of DCFH-DA (S0033, Beyotime, China) at 37°C for 20 min. Rosup-treated cells were used as positive control. Fluorescence images of cells were visualized by the Leica MZ10F modular stereomicroscope to determine the intracellular ROS generation.

### Mitochondrial Membrane Potential (Δψm)

Mitochondrial membrane potential (ΔΨm) was detected by the mitochondrial membrane potential assay kit (C2006, Beyotime, China) (Xiao et al., 2017). Cells were exposed to PA/OA (200 μM) with or without various concentrations of Cori (10, 20 µM) for 24 h. After washing with JC-1 staining buffer, the cells were suspended in PBS buffer. Cccp-treated cells were used as a positive control. The cells were then incubated with10 µM JC-1 at 37°C for 20 min in the dark. The fluorescence intensity was observed within 30 min under the fluorescence microscope (Leica).

### Extraction of Total mRNAs and Quantitative Real-Time PCR (RT-PCR)

Total mRNAs from the liver and AML12 cells were extracted by Trizol reagent (Invitrogen, CA, USA). The mRNAs were extracted, reverse-transcribed into complementary DNA (cDNA) according to the manufacturer’s protocols (TaKaRa, Otsu, Japan). Quantitative real-time PCR was performed using SYBR Premix ExTaq (TaKaRa, Otsu, Japan) on ABI 7500 Fast Real-Time PCR System (Applied Biosystems, Foster City, CA, USA). The relative expression level of each sample was analyzed by the 2^−ΔΔCt^ method, using GAPDH as a reference gene. Primers pairs are listed in **Table 2**.

**Table 2:** Primer Sequences for real-time PCR (RT-PCR) analysis.

Primer	Forward	Reverse
FASN	AGCACTGCCTTCGGTTCAGTC	AAGAGCTGTGGAGGCCACTTG
ACC1	GAAGTCAGAGCCACGGCACA	GGCAATCTCAGTTCAAGCCAGTC
SREBP1-c	CTCCGGCCACAAGGTACACA	GAGGCCCTAAGGGTTGACACAG
PPARα	TCCTGAGCCATGCAGAATTTAC	AGTCTAAGGCCTCGCTGGTG
CPT1α	GGAATGAAATTCCCACTGTCTGTC	CAGTTCAGCCATCGCTGTTGTA
ACOX-1	CGGAAGATACATCCCGGAGACC	AAGTAGGACACCATACCACCC
MCP1	AGGTCCCTGTCATGCTTGTG	TCTGGACCCATTCCTTCTTG
F4/80	CTTTGGCTATGGGCTTCCAGTC	GCAAGGAGGACAGAGTTTATCGTG
TNF-α	TGGGCCTCTCATGCACCACC	GAGGCAACCTGACCACTCTCCCT
IL-6	AGACAAAGCCAGAGTCCTTCAG	GCCACTCCTTCTGTGACTCCAG
COXII	GCCGACTAAATCAAGCAACA	CAATGGGCATAAAGCTATGG
NRF1	CAACAGGGAAGAAACGGAAA	GCACCACATTCTCCAAAGGT
NRF2	TAGATGACCATGAGTCGCTTGC	GCCAAACTTGCTCCATGTCC
TFAM	GGGAAGAGCAAATGGCTGAA	TCACACTGCGACGGATGAGA
GAPDH	TGAACGGGAAGCTCACTGG	TCCACCACCCTGTTGCTGTA

### Western Blot Analysis

Protein was extracted from AML12 cells or liver tissues with RIPA buffer (50 mM Tris HCl, pH 8.0, 150 mM NaCl, 5 mM EDTA, 1% Nonidet P-40, 0.5% sodium deoxycholate, 1 mM PMSF, 1 mM NaVO4, 1 mM NaF, 0.1% SDS), and protease inhibitor cocktail (Roche, Germany). Immunobloting was then performed as described previously (Shin et al., 2019). In brief, lysates were centrifuged (13,000 × g for 15 min) and the supernatant was collected for the following protein quantification using BCA Protein Assay Kit (Pierce). Protein extracts were separated on 12% or 15% SDS-PAGE gels and electrophoretically transferred onto polyvinylidene fluoride (PVDF) membrane. After blocking, the membranes were blocked with 5% nonfat dry milk for 1 h at room temperature, followed by immunostaining with primary antibodies against target proteins at 4°C overnight. Immunoreactive proteins were stained with ECL Detection Kit (Amersharm Pharmacia, UK). Protein expression levels were then determined by analyzing the signals captured on PVDF membranes by using the Chemi-doc image analyzer (Bio-Rad, Hercules, CA). The antibodies were listed as follows (LC3A/B at 1:1,000 dilution, CST; Parkin at 1:500 dilution, Abcam). GAPDH (at 1:1,000 dilution, CST) was used as an internal standard.

### Oxygen Consumption Rate

The AML12 cells were seeded in XF24 cell culture microplates. After overnight culture, the cells were incubated with PA/OA in the presence or absence of Cori (10, 20 μM) for 24 h. OCR measurements were then carried out according to the Seahorse assay protocol. Basal OCR is [OCR with substrates - OCR with rotenone and antimycin A]. Maximal respiratory capacity is [OCR with FCCP - OCR with rotenone and antimycin A] (Sinha et al., 2015).

### Statistical Analysis

Data were pooled and represented as mean ± SD. The statistical significance of differences was determined using either the Student’s t test (2-tailed) or One-way ANOVA followed by Bonferroni’s multiple comparison *post hoc* tests. A value of *p* < 0.05 was considered statistically significant.

## Data Availability Statement

All datasets generated for this study are included in the article/supplementary files.

## Ethics Statement

All the animal experiments were approved by the ethics committee of the Ninth People’s Hospital, Medical School of Shanghai Jiaotong University, Shanghai, China (project No. SH9H-2016-TK356-1) and conducted according to the guidelines for the Care and Use of Laboratory Animals.

## Author Contributions

Conceptualization and project administration: RZ, GW, and ML. Resources: ML. Data curation, formal analysis, investigation, software, visualization, writing original draft, and writing review or editing: RZ, GW. Methodology, supervision, and validation: RZ, KC, NZ, JW, LM, CZ, XC, GW, and ML.

## Funding

This research was funded by the National Natural Science Youth Foundation of China (No. 81503579), the Medical and Engineering Integration Project of Shanghai Jiaotong University (No. YG2016MS07), the Project funded by China Postdoctoral Science Foundation (No. 2017M621449), the Xiamen Science and Technology Bureau Project (No. 3502Z20189011, No. 3502Z20184013), the National Key R&D Program of China (No. 2018YFC1704400), and the Science and Technology Commission of Shanghai Municipality (No. 15401932900, No. 16401933200).

## Conflict of Interest

The authors declare that the research was conducted in the absence of any commercial or financial relationships that could be construed as a potential conflict of interest.

## Abbreviations

ACC1, acetyl-CoA carboxylase 1; ACOX1, peroxisomal acyl-CoA oxidase 1; AML12, hepatocyte cell line alpha mouse liver 12 cells; Cori, Corilagin; CPT1α, carnitine palmitoyltransferase 1α; FASN, fatty acid synthase; FFA, free fatty acid; HFD, high-fat diet; IL-6,interleukin-6; LC3, microtubule-associated protein 1 light chain 3; MCP1, monocyte chemoattractant protein-1; MMP (ΔΨm), mitochondrial membrane potential; MRC, mitochondrial respiratory chain; mtDNA, mitochondrial DNA; mtFAO, mitochondrial fatty acid oxidation; NAFLD, nonalcoholic fatty liver disease; NASH, nonalcoholic steatohepatitis; NCD, normal chow diet; OCR, oxygen consumption rate; PPAR-α, peroxisome proliferator activated receptor-α; p62/SQSTM1, sequestosome 1; ROS, reactive oxygen species; SREBP-1c, sterol regulatory element binding protein-1c; TEM, transmission electron microscopy; TNF-α, tumor necrosis factor-α; VDAC1, voltage-dependent anion channel 1; 3-MA, 3-methyladenine; 8-OHdG, 8 hydroxydeoxyguanosine.
